# Does planning to mixed feed undermine breastfeeding?

**DOI:** 10.1111/mcn.13610

**Published:** 2023-12-13

**Authors:** Stamatia Michalopoulou, Ada L. Garcia, Linda Wolfson, Charlotte M. Wright

**Affiliations:** ^1^ Human Nutrition, School of Medicine, Dentistry and Nursing University of Glasgow Glasgow Scotland UK; ^2^ Improving Health and Wellbeing Scottish Government Glasgow Scotland UK

**Keywords:** breastfeeding, breastfeeding support, formula feeding, maternal and infant feeding survey

## Abstract

Continued breastfeeding is important for infants' health, but it is unclear whether mixed feeding increases the risk of breastfeeding cessation. We aimed to explore associations of mixed feeding and lactation problems with early cessation of breastfeeding. We analysed data from mothers who completed the Scottish National Maternal and Infant Feeding Survey and had previously breastfed their infants. At age 8–12 weeks, mothers (*N* = 1974) reported their feeding history and intentions, lactation problems and reasons for giving formula milk. The main outcome measure was cessation of breastfeeding before 6–8 weeks and time to cessation. By 6 weeks, 65% had mixed fed at some point, 32% had ceased breastfeeding, 22% were currently mixed feeding and 46% were exclusively breastfeeding. Lactation problems before 2 weeks were common (65%), and strongly associated with stopping breastfeeding (relative risk [RR]: 3.23, 95% confidence interval [CI]: 2.0–5.3) and with mixed feeding (RR: 3.14, 95% CI: 2.5–4.0). However, even after adjustment for breastfeeding problems mothers who planned to mixed feed (RR: 3.39, 95% CI: 2.4–4.9) and those who introduced formula for practicalities (RR: 3.21, 95% CI: 2.3–4.4) were more likely to stop breastfeeding. These variables also predicted later lactation insufficiency (planned mixed feeding RR: 1.39, 95% CI: 1.0–2.0; formula for practicalities RR: 1.76, 95% CI: 1.3–2.3). Mothers who received specialist lactation support were less likely to cease breastfeeding (RR: 0.63, 95% CI: 0.5–0.9) but nonspecialist input was unrelated to risk of cessation (RR: 1.06, 95% CI: 0.2–4.9). In conclusion, choosing to mix feed an infant is strongly associated with stopping breastfeeding, even in the absence of lactation problems.

## Key messages

- According to the Scottish Maternal and Infant Feeding Survey, most mothers did not intend to mixed feed but a large proportion eventually mixed fed at some time.
- Lactation problems were commonly present during the first 2 weeks after birth, and those mothers who initially planned to mix feed were more likely to have had early and late lactation problems compared to mothers who maintained exclusive breastfeeding.
- After adjusting for early problems, mothers who planned to mixed feed or introduce formula were more likely to stop breastfeeding than those who did not.
- Specialist breastfeeding support was associated with a reduced risk of breastfeeding cessation.

## Graphical abstract

Mixed feeding of formula milk with breastfeeding is associated with feeding problems and with stopping breastfeeding, but mothers who planned to mixed feed or use formula for practical reasons were more likely to go on to have milk insufficiency and to stop breastfeeding compared to exclusive breastfeeders.

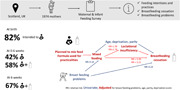

## 1 INTRODUCTION

The importance of breastfeeding for both infant and mother's health are irrefutable, with the most impact via infection risk reduction (Horta & Victora, 2013; Kramer & Kakuma, 2012; Victora et al., 2016). Exclusive breastfeeding before 6 months is important both to avoid risk of infection via contaminated drinks, but also because consumption of other energy rich (nutritive) drinks such as formula milk (mixed feeding) will displace breast milk. This will both reduce its protective effect and potentially lead to secondary lactation failure, as a continued milk supply depends upon regular suckling. It has been well shown that neonatal introduction of formula milk increases the risk of exclusive breastfeeding cessation and shorter breastfeeding duration (Pérez‐Escamilla et al., 2022). A report by UNICEF and WHO indicated that timely introduction of breast milk rates were twice as high among infants who received solely breast milk compared with infants who received formulas in the first 3 days of life (UNICEF WHO, 2018).

Self‐reported milk insufficiency is one of the most common reasons why mothers introduce formula milk (Segura‐Pérez et al., 2022). However, it is not clear whether the lactation failure the mothers report is related to perceived or actual inadequate milk quantity and quality (Huang et al., 2022; Segura‐Pérez et al., 2022). Evidence from a systematic review of 120 studies shows that the risk factors for lactation problems include maternal psychosocial characteristics (e.g., age, education, employment status, income, parity, BMI), delivery practices (e.g., caesarean delivery, reduced breastfeeding practices in the maternity ward), breastfeeding practices (e.g., no intention to breastfeed, low breastfeeding support, previous experience of breastfeeding difficulties) and baby behaviours (e.g., unsettled infant, feeding difficulties) (Segura‐Pérez et al., 2022). Often unsettled infant behaviours are misinterpreted by the parents, who believe that these are signs of digestive problems, allergies or hunger and insufficiency of milk (Cook et al., 2019; Mohebati et al., 2021) and lead them to formula supplementation as a perceived solution (Vilar‐Compte et al., 2022).

It has been known for some time that giving formula early in life greatly increases the risk of breastfeeding cessation (Pérez‐Escamilla et al., 2022). However, it is less clear whether mixed feeding itself causes later breastfeeding cessation, via secondary lactation failure, or whether it simply reflects the process of breastfeeding cessation, either because of other lactation problems or for other reasons, such as return to work. It has also been suggested that expectations around breastfeeding exclusivity are unrealistic, and some practitioners may recommend mixed feeding in the hope that it may encourage women to breastfeed for longer (Hoddinott et al., 2012). An analysis is thus needed that can separate the influences of mixed feeding per se, from the effects of early breastfeeding problems.

Using an existing population survey we aimed to investigate (1) The reasons women give for adopting mixed feeding of breast and formula milk, (2) The extent to which choosing to mixed feeding is associated with an increased risk of breastfeeding cessation, and with later lactation failure and (3) The extent to which support from health staff relates to the use of mixed‐feeding and continued breastfeeding.

## 2 METHODS

### 2.1 Study design

This is a secondary analysis of the Scottish Maternal and Infant Feeding Survey, a population representative survey about attitudes, feeding choices and previous actions of pregnant women and new parents resident in Scotland (Scottish Government, 2018).

### 2.2 Data collection

The survey comprised three different cross‐sectional samples of pregnant women and mothers of infants aged 8–12 weeks and 8–12 months. Around 2500 women in each group were asked to complete a questionnaire between March and July 2017. It is the data from the 8–12 weeks questionnaire, which are used in this paper.

For the 8–12 weeks wave, National Birth Registration Records were used to identify eligible infants and their mothers who had given birth in and lived in Scotland between 1st March and 30th April 2017. Women or infants who were known to have died were excluded. Survey packs were mailed to all eligible mothers when infants were aged 6–9 weeks and a reminder was sent after 3 weeks to mothers who had not responded (Scottish Government, 2017).

The questionnaire included detailed questions on breastfeeding since birth and this study concerns the mothers in that sample who had ever given their baby breast milk. The questionnaire included questions about feeding practice at 6–8 weeks, on whether they had stopped breastfeeding and if so when, and their original intention regarding mixed feeding. Respondents were also presented with a range of different possible lactation problems or situations and rated the extent they applied to them. Those who had ceased breastfeeding were asked to rate the applicability of several statements about reasons for cessation, while those who had ever given formula milk were asked to rate the applicability of several statements about why they had done this.

### 2.3 Data preparation and outcomes

The survey answers were entered and cleaned by the survey team and supplied in anonymized form to the research team.

Ever mixed feeding was defined as having given both breast and formula milk at some point in the past 6 weeks and was identified by asking mothers whether they had ever given formula milk as well as breast milk and the age it was first given. However, the amount of formula given was not recorded, nor when it was given regularly, so this group will have included babies who had been given formula shortly after birth, but who may not have continued and may by 6 weeks have been exclusively breastfed. However, this group could be split into those who reported retrospectively that they had intended to mixed feed (Planned Mixed Feeding) and those who had not intended to do so (Unplanned Mixed Feeding). In addition, mothers reported whether they were still mixed feeding at child's age 6–8 weeks, that is still breastfeeding, but also giving formula at least a few times per week. Exclusive breastfeeding at 6 weeks was thus defined as reported feeding of breast milk only at that time.

The outcomes of interest were age at breastfeeding cessation, continuance of breastfeeding to age 6 weeks and late lactation insufficiency, with mixed feeding, breastfeeding problems and exposure to professional help and socioeconomic status as potential predictors.

To avoid multiple significant tests and reduce the likelihood of Type‐1 errors, before analysis the questions on mixed feeding intentions, reasons for formula feed introduction and breastfeeding cessation were combined into broad categories, based on the type of reason given (Supporting Information: Table S1). Each situation/condition was defined as being present if they answered yes to any of those items.

The reasons why breastfeeding mothers introduced formula feeds were divided into four groups: Breastfeeding problems, Professional advice, Practicalities, and Perceived milk insufficiency. The reasons for breastfeeding cessation were categorised into four groups: Personal choice/convenience, Dislike/lack of confidence, Perceived lactation insufficiency and other breastfeeding problems (e.g., they found breastfeeding too difficult or had feeding problems) (Supporting Information: Table S1).

Lactation problems were assessed at three different time points (at the maternity unit, within the first 2 weeks at home and between 2 and 6 weeks). The first two categories were combined into ‘early’ and the last one indicated ‘late’ breastfeeding problems. The range of problems were combined into three categories, as unrelated, possibly related and definitely related to milk insufficiency (Supporting Information: Table S1). Late lactational insufficiency was assessed as a possible outcome of mixed feeding, while analyses were adjusted for early lactational insufficiency.

Mothers were asked whether they had received support and advice on breastfeeding from a wide range of professionals. The sources of support were categorised into two groups: generic (midwives, health visiting team and general practitioners) and specialist (lactation counsellors, National Health Service [NHS] infant feeding advisors, tongue tie clinic, lactation support workers).

Maternal sociodemographic characteristics included maternal age, parity, ethnicity, and a proxy of deprivation defined by the Scottish Index of Multiple Deprivation (SIMD). SIMD is expressed in quintiles, with 1 referring to the most deprived and 5 to the least deprived. Information on the educational level was not collected.

### 2.4 Statistical analysis

All statistical analyses were performed using IBM SPSS Statistics 23. Logistic regression models were used to evaluate whether planned mixed feeding or introducing formula for practicalities increased the likelihood of either breastfeeding cessation at 6 weeks or of late milk insufficiency. Crude and adjusted models were conducted, and the relative risks (RRs) with 95% confidence intervals (CIs) were calculated.

To further evaluate the effect of Planned Mixed Feeding compared to Unplanned Mixed Feeding on time to breastfeeding cessation, survival analysis was performed. Those who had never mixed fed were, by definition, still breastfeeding. The time variable of the models was the age in weeks when the mothers last breastfed their infants. Those that had ceased were coded as 0 and were the event cases and those that did not, were coded as 1 and were the censored cases. Due to the selection criteria used, no participants were left before the end of the study.

The log‐rank test was applied to examine whether breastfeeding duration differed between the two subgroups of feeding practices. Subsequently, the risk of breastfeeding cessation at 6 weeks due to mixed feeding practices was estimated using Cox's proportional hazards models. Crude and adjusted hazard ratios (HRs) with 95% CI were calculated.

### 2.5 Ethical statement

The survey was approved first by the Public Benefit and Privacy Panel for Health and Social Care and then received approval by NHS Scotland in December 2016 (Scottish Government, 2017).

## 3 RESULTS

### 3.1 Study population and infant feeding characteristics

**Figure 1:**
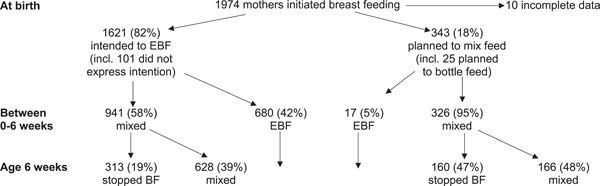
Flow diagram of number and proportion of the feeding practices at birth, at 0–6 weeks and at 6 weeks of age. BF, breastfeeding; EBF, exclusive breastfeeding.

A total of 1974 mothers had initiated breastfeeding and were included in the study. The majority were either White Scottish (65%) or White British (12%) or other White (13%) with only 5% of South Asian origin and 3% other ethnic groups. Most participants (82%) indicated that they did not intend to mix feed, but 65% did eventually mixed feed at some point. At 6–8 weeks, 40% of mothers overall were still exclusively breastfeeding, 24% were mixed feeding and 36% were offering solely formula feeds (Figure 1). Older and less deprived mothers were less likely to have ever mixed fed compared to those who were younger or more deprived. Primiparous mothers were less likely to plan to mixed feed but more likely actually have done so (Supporting Information: Table S2).

### 3.2 Reasons given for mixed feeding and associations with lactation insufficiency and breastfeeding cessation

The most common reasons given for starting mixed feeding were the presence of lactation problems (30%) and practicalities (26%), while the main reasons for breastfeeding cessation were lactation problems (57%) and perceived milk insufficiency (45%).

The prevalence of any lactation problems was higher during the first 2 weeks compared to later. All mixed feeders were much more likely to report early, and late problems compared to those exclusively breastfeeding (Table 1). Unplanned Mixed Feeding had the highest rates of breastfeeding problems before and beyond 2 weeks, with those strongly related to lactational insufficiency being the most common (Table 1). Early lactation problems were strongly related to stopping breastfeeding by the second week, compared to those that did not face any early problems (RR: 3.23, 95% CI: 2.0–5.3). On the other hand, late lactation problems were not significantly associated with breastfeeding cessation by the sixth week (RR: 1.22, 95% CI: 0.9–1.6). Mothers who planned to mixed feed and those who introduced formula for practicalities were much more likely to stop breastfeeding at 6 weeks compared to those that did not, even after adjustment for sociodemographic factors, and breastfeeding problems (Table 2).

**Table 1:** Number and prevalence of breastfeeding problems according to mixed feeding category.

	Before 2 weeks			Beyond 2 weeks		
	*N* a	%	RR (95% CI) of problems compared to EBF	*N*	%	RR (95% CI) of problems compared to EBF
Any kind of breastfeeding problem						
Whole sample	1185	64.8		784	46.1	
EBF	333	47.8	Ref	240	34.4	Ref
Unplanned mixed feeding	668	77.0	3.29 (2.7–4.1)	447	58.0	2.63 (2.1–3.2)
Planned mix feeding	155	61.0	1.54 (1.5–2.1)	90	40.9	1.32 (0.97–1.8)
Problems possibly related to lactational insufficiency: Baby does not suck, was sleepy, unsettle, had frequent feeds						
Whole sample	540	28.4		489	28.8	
EBF	117	16.8	Ref	137	19.7	Ref
Unplanned mixed feeding	350	38.2	3.06 (2.4–3.9)	293	38.0	2.51 (2.0–3.2)
Planned mixed feeding group	67	24.2	1.58 (1.1–2.2)	53	24.1	1.30 (0.9–1.9)
Problems strongly related to lactational insufficiency: Not enough milk production, weight loss, very slow weight gain						
Whole sample	775	42.3		364	21.4	
EBF	153	22.0	Ref	55	7.9	Ref
Unplanned mixed feeding	499	57.5	4.81 (3.8–6.0)	248	32.2	5.54 (4.0–7.6)
Planned mixed feeding	115	45.3	2.94 (2.2–4.0)	55	25.0	3.89 (2.6–5.9)

Abbreviations: CI, confidence interval; EBF, exclusive breastfeeding; RR, relative risk.

^a^ Total numbers between whole sample and sum of sub‐categories differ due to missing data.

**Table 2:** Crude and adjusted models for breastfeeding cessation and late milk insufficiency at 6 weeks predicted by planned mixed feeding and formula introduction due to practicalities.

Outcome	Crude model RR (95% CI)	Adjusted model^a^ RR (95% CI)
Breastfeeding cessation at 6 weeks		
Predictors		
Mixed feeding planned before birth	3.20 (2.5–4.1)	3.39 (2.4–4.9)
Formulas for practicalities	3.10 (2.5–3.9)	3.21 (2.3–4.4)
Late milk insufficiency		
Predictors		
Mixed feeding planned before birth	1.25 (0.9–1.7)	1.39 (1.0–2.0)
Formulas for practicalities	1.55 (1.2–2.0)	1.76 (1.3–2.3)

Abbreviations: CI, confidence interval; RR, relative risk; SMID, Scottish Index of Multiple Deprivation.

^a^ Adjusted for maternal age, parity, and SIMD, early lactational problems and early problems unrelated to milk insufficiency.

Planned mixed feeding was not significantly associated with reported later lactational sufficiency in univariate analysis, but after adjustment it was associated with a 39% increased risk. Formula feed introduction for practicalities was associated with 55% increased risk for later lactational problems, which increased to 76% after adjustment (Table 2).

**Figure 2:**
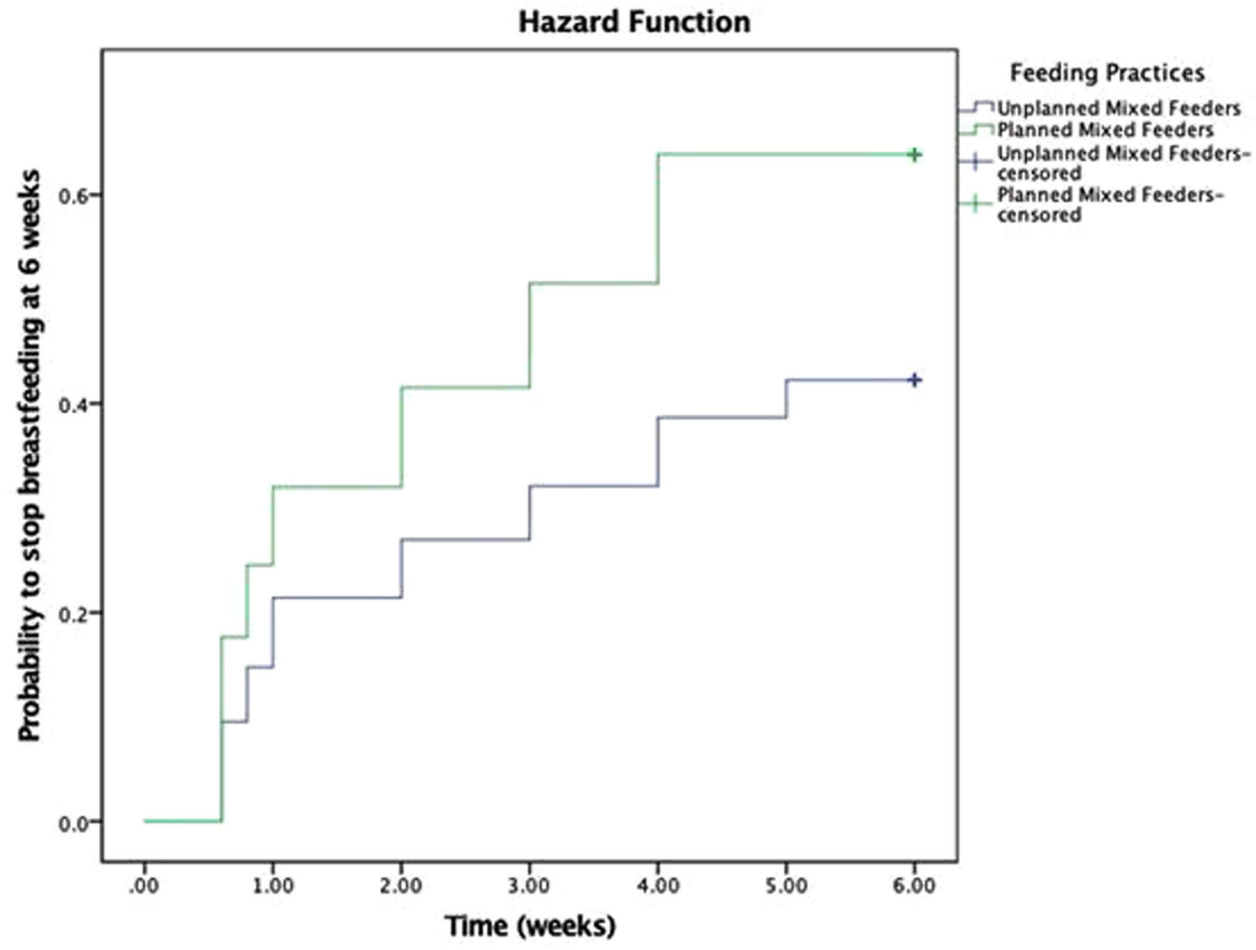
Survival function of time to breastfeeding cessation by whether planned to mixed feed.

Kaplan–Meier curves (Figure 2) demonstrated that the Planned Mixed Feeding group tended to stop breastfeeding earlier than the Unplanned group. Adjustment for maternal age, parity, deprivation, early and late lactational problems using Cox's proportional hazards modelling, gave an HR for Unplanned Mixed Feeding compared to Planned Mixed Feeding 0.57 (95% CI:0.4–0.8).

### 3.3 Health staff support

Almost all mothers reported that they had access to generic staff support (*N* = 1194, 98%), while 38% (*N* = 466) accessed specialist lactation help. Only specialist help was associated with a reduced risk of quitting breastfeeding. Specifically, after adjustment for age and early breastfeeding problems, mothers who received specialist help had 28% decreased risk of stopping breastfeeding at 6 weeks, compared to mothers that did not receive any staff help (Table 3). Staff help, either generic or specialist, was not associated with mixed feeding at 6 weeks (data not shown).

**Table 3:** Crude and adjusted models for breastfeeding cessation at 6 weeks as predicted by whether received any generic or specialist help.

Predictor	Outcome: Breastfeeding cessation at 6 weeks		
	Crude model RR (95% CI)	Model 1^a^ RR (95% CI)	Model 2^a^ RR (95% CI)
Generic help	1.01 (0.4–2.8)	0.70 (0.6–0.8)	0.93 (0.3–2.9)
Specialist help	0.52 (0.4–0.7)	0.53 (0.4–0.7)	0.72 (0.6–0.9)

Abbreviations: CI, confidence interval; RR, relative risk.

^a^ Model 1 = adjusted for maternal age, Model 2 = Model 1 + early breastfeeding problems.

## 4 DISCUSSION

The study revealed that while most mothers planned to exclusively breastfeed, two‐thirds in fact gave some formula during the first 6 weeks. The majority of mothers in this study described themselves as having some sort of perceived problem with breastfeeding and the number of problems was strongly related to both mixed feeding and early breastfeeding cessation. While many studies have described insufficient milk supply as a main reason for formula feed introduction (Segura‐Pérez et al., 2022), the use of formula as cause of insufficiency has been less studied. There is strong trial evidence to show that early introduction of solids results in down regulation of breast milk intake (Cohen et al., 1994) and it has been shown in observational studies that once formula feeds are introduced, breastfeeding frequency is reduced, resulting in less milk production (Pérez‐Escamilla et al., 2023) and reduction in the amount of milk removed during a feed (Prime et al., 2012). It thus seems highly likely that this leads to suppression of milk production and insufficient milk supply. This phenomenon has been well observed in the neonatal period (Pérez‐Escamilla et al., 2022) and an earlier UK Infant Feeding study found an association between mixed feeding and cessation in the early weeks (McAndrew et al., 2012) but was not able to separate the potential confounding influence of breastfeeding problems, with the introduction of formula potentially simply representing the beginning of breastfeeding cessation. We thus planned an analysis to consider the effect of mixed feeding in the absence of breastfeeding problems.

While 30% of mothers attributed their use of formula to breastfeeding problems, 26% of mothers supplemented at least partially for practical reasons and 18% had planned from the outset to mixed feed. This analysis thus examined whether those who initially planned to mixed feed and those who started mixed feeding for practicalities were more or less likely to stop breastfeeding and found a three times higher risk for early breastfeeding cessation by age 6 weeks, even after adjustment for the extent to which they had experienced lactation problems.

We found that both mixed feeding and early cessation were related to younger maternal age and greater deprivation, as has been observed in many previous studies in developed societies (Wright et al., 2006). A perception that breast milk is insufficient has been cited by around half of mothers worldwide (Pérez‐Escamilla et al., 2023; Segura‐Pérez et al., 2022) and it has been suggested that most of these ‘problems’ represent normal infant behaviour (Pérez‐Escamilla et al., 2023), but that the formula milk industry has exploited these issues and now promotes its products as their solution, despite a lack of evidence for this (Rollins et al., 2023). Primiparous mothers were also more likely to actually mixed feed, as also described in other studies (Pérez‐Escamilla et al., 2023), but it was multiparous mothers who were more likely to have planned to mixed feed, which is concerning. Another effect of formula milk marketing is to present breast milk substitutes as equivalent to and as effective as breastfeeding, which may be why some mothers adopt mixed feeding even in the absence of difficulties (Pérez‐Escamilla et al., 2023). Previous smaller studies have evaluated plans for mixed feeding in relation to quitting breastfeeding. A Brazilian cohort study found that 57% of the 374 mothers planned to mix feed and 22% planned to do so in the first month, but reported only on all women who actually initiated mixed feeding in the first month, showing that they had a shorter duration of breastfeeding, compared to those starting later (65 vs. 165 days, respectively, *p* < 0.001) (Marques et al., 2001). Another prospective cohort study of 74 primiparous women found that mothers who planned to mix feed had significantly shorter breastfeeding duration, compared to those that planned to solely breastfeed (10.2 vs. 18.5 weeks respectively, *p* = 0.004) (Chezem et al., 2003).

The quantity and quality of help and support that mothers receive is an important factor in sustaining breastfeeding; a review of 100 randomised trials found that any kind of support, specialist or generic, was effective to delay cessation of exclusive or partial breastfeeding cessation (McFadden et al., 2017). In the present study, most mothers had access to some sort of breastfeeding support, but it was specialist support that was associated with longer duration of breastfeeding. It could be speculated that those mothers who planned to mixed feed might be less likely to seek support, but we found that neither specialist nor generic help related to mixed feeding at 6 weeks. Thus, the beneficial effect of specialist input seems unlikely to have operated via the mechanism of discouraging mixed feeding.

### 4.1 Strengths and limitations

The strength of this analysis is that it draws on recent data from a population representative survey and included large numbers of breastfeeding mothers. However, the survey was conducted in 2017 so it might not reflect changes in infant milk feeding choices during the Covid‐19 pandemic and more recently with cost‐of‐living crisis and the sharp increase in formula price. The more recent rates of breastfeeding in Scotland show that by the age of 6–8 weeks, 32% of infants were exclusively breastfeeding and 14% were mixed feeding (Scottish Government, 2023), this shows a small reduction in exclusive breastfeeding rates compared to the data reported in 2017 but an increase in mixed feeding prevalence and highlights the dynamic nature of population surveys.

By adjusting for all reported problems, this analysis allows the influence of mixed feeding itself to be isolated from the factors that lead to it being adopted. However, there were no data about when exactly babies were first given formula regularly, and the follow up period was so short, so we could not examine the extent to which any mixed feeding related to breastfeeding cessation. The UK Infant Feeding Survey was similarly unable to detect when children began to be regularly mixed fed (McAndrew et al., 2012). However, by picking out those mothers who chose to mixed feed in advance, or for practicalities, we have been able to explore whether mixed feeding itself predisposes to cessation, as opposed to it being a result of breastfeeding problems.

This was an analysis of mainly cross‐sectional data with recall only of earlier issues and intentions, which may have introduced recall bias. A further limitation is that we have data only up to age 6 weeks, so this analysis is not informative about the impact of mixed feeding on continued breastfeeding beyond that age. A prospective study could provide more robust data on antenatal intentions and the exact time that formulas were introduced for the first time, but these are challenging to mount at sufficient scale to be informative.

In this analysis, the association of planned mixed feeding with insufficiency was less strong than the association with cessation, so it is unlikely to fully explain this. However, as we could record only perceived, rather than actual milk insufficiency, and the classification of the lactation problems into three categories may not be robust, it may be that this outcome has been incompletely captured.

This analysis had a deliberately narrow focus and could not address the wider range of possible influences on feeding and due to the observational nature of the findings, we cannot infer causality. However, a trial which actively discouraged mixed feeding as well as supporting breastfeeding would help to clarify this, as well as having the potential to adjust for all the other possible factors that may support or undermine breastfeeding.

### 4.2 Implications

Perceived problems with breastfeeding are common and current commercial influences encourage mothers to see the introduction of formula milk as a solution. The role of formula companies in fuelling these worries and suggesting that their products have the potential to cure these, usually without objective scientific evidence, has been described in detail recently (Rollins et al., 2023) as well as a call for tighter regulation on the claims that formula milk companies can make for their products (Baker et al., 2023).

While primary care staff now generally recognise the importance of breastfeeding, they still often lack the skills needed to give parents practical advice and the confidence to explain the dangers of mixed feeding (Pérez‐Escamilla et al., 2023). We have recently been reminded of the need for better training for health staff on how to assess and address perceived milk insufficiency and the risks of formula milk (Baker et al., 2023). This analysis provides further evidence to support this view and underlines the need for staff to fully understand and be able to explain the risks of mixed feeding. In particular, health staff need to understand that supplementation undermines rather than sustaining breastfeeding.

## 5 CONCLUSIONS

Planned mixed feeding and use of formula for practical reasons is strongly independently associated with cessation, suggesting that mixed feeding does indeed lead to secondary lactation failure, or simply a lower overall commitment to breastfeeding.

## AUTHOR CONTRIBUTIONS

Stamatia Michalopoulou undertook the analyses and produced the first draft. Ada L. Garcia formulated the research questions, advised on data analysis and supervised the initial write up. Linda Wolfson planned and ran the original survey, helped plan the analysis and commented on the initial results. Charlotte M. Wright sat in the steering group of the original survey, formulated the research questions and advised on data analysis and initial write up. She then reworked the final draft. All authors approved the final manuscript as submitted and agreed to be accountable for all aspects of the work.

## CONFLICT OF INTEREST STATEMENT

The authors declare no conflict of interest.

## Supporting information

Supporting information.

## Data Availability

The data that support the findings of this study are openly available in The National Archives, UK Open Government Licence v3.0. at https://www.gov.scot/publications/scottish-maternal-infant-nutrition-survey-2017/documents/.
