# Supplementary material for: Does planning to mixed feed undermine breastfeeding?
Source: Matern Child Nutr. 2023 Dec 13;20(2):e13610. doi: 10.1111/mcn.13610 (PMC10981487; doi:10.1111/mcn.13610)
Supplement: Supplementary file 1 — Supporting information. [file MCN-20-e13610-s001.docx]

**Supplementary Material**

**Table S 1** Combination of individual variables into scores

| **Reasons for formula feeds introduction** | **Variables combined** | **% *** |
| --- | --- | --- |
| Breastfeeding problems | I had breastfeeding problems | 30.1 |
| Practicalities | I always intended to mix feed; It allowed my partner to be involved; To make breastfeeding more manageable; To help my baby sleep longer; I was attending a social event and it was easier to give formula | 26.4 |
| Concerns for milk insufficiency | I was anxious about how much milk by baby was getting and decided to give formula supplement | 21.7 |
| Professional advice | A health professional advised me (e.g. nurse, doctor, midwife or health advisor) | 20.2 |
| **Reasons for giving up breastfeeding** |  |  |
| Breastfeeding problems | I found it too difficult; I had feeding problems | 56.6 |
| Perceived milk insufficiency | I didn’t think the baby was getting enough milk | 45.3 |
| Dislike, embarrassment, lack of confidence | I didn’t like breastfeeding; I didn’t like using breast pump; I was embarrassed feeding in front of people, I didn’t feel confident | 25.3 |
| Choice, external factors | I breastfed/expressed milk for as long or longer than I intended to; I returned to work; I was too tired | 22.8 |
| **Breastfeeding problem categories** |  |  |
| Unrelated to milk insufficiency | My baby was premature, small or unwell,  My baby wouldn’t attach at the breast, I had difficulty attaching the baby to the breast myself,  My baby had a tongue tie that was causing problems,  I had full or engorged breasts, I had sore nipples, I had sore breasts,  I had mastitis | 64.4 |
| Possible milk insufficiency | My baby attached, but wouldn’t suck, My baby was sleepy and wouldn’t feed, My baby wouldn’t settle after feeds, Prolonged or frequent feeds | 52.9 |
| Definite milk insufficiency | I didn’t produce enough milk, I was worried about how much milk my baby was getting,  My baby lost a lot of weight, My baby’s weight gain was very slow | 47.6 |

*** Answered yes to one or more items**

***Table S2*** *Main characteristics of the participants, broken down by whether ever mixed fed or planned to mixed feed*

|  | **All** | | **% Ever**  **Mixed, within category** | | **P χ^2^** linear  **Compared to never** | **% Planned**  **Mixed, within category** | | **P χ^2^**  **Compared to not** |
| --- | --- | --- | --- | --- | --- | --- | --- | --- |
| **Maternal Age (years)** | **N** | **(%)** | **N** | **(%)** |  | **N** | **(%)** |  |
| ≤19 | 18 | 0.9 | 15 | 83.3 | 0.017 | 3 | 16.7 | 0.796 |
| 20-24 | 92 | 4.7 | 74 | 80.4 |  | 14 | 15.2 |  |
| 25-29 | 418 | 21.2 | 278 | 66.5 |  | 73 | 17.5 |  |
| 30-34 | 784 | 39.8 | 478 | 61.0 |  | 134 | 17.1 |  |
| ≥35 | 660 | 33.5 | 429 | 65.1 |  | 120 | 18.2 |  |
| **Scottish Index of Multiple Deprivation** | | |  |  |  |  |  |  |
| 1=Most deprived | 254 | 12.9 | 170 | 66.9 | 0.003 | 45 | 17.7 | 0.528 |
| 2 | 307 | 15.6 | 210 | 68.6 |  | 49 | 16.0 |  |
| 3 | 398 | 20.2 | 275 | 69.1 |  | 71 | 17.8 |  |
| 4 | 491 | 24.9 | 309 | 63.1 |  | 80 | 16.3 |  |
| 5=Least Deprived | 524 | 26.5 | 311 | 59.4 |  | 100 | 19.1 |  |
| **Parity** |  |  |  |  |  |  |  |  |
| Multiparous | 981 | 49.7 | 578 | 59.0 | <0.001 | 207 | 21.1 | 0.001 |
| Primiparous | 991 | 50.3 | 696 | 70.3 |  | 138 | 13.9 |  |
| **Presence of breastfeeding/expressing breast milk problems** | | |  |  |  |  |  |  |
| Yes | 1349 | 68.7 | 767 | 56.8 | <0.001 | 206 | 15.3 | 0.84 |
| No | 615 | 31.3 | 188 | 30.0 |  | 96 | 15.6 |  |
